# Effect of intermittent pneumatic compression on preventing deep vein thrombosis using microfluidic vein chip

**DOI:** 10.3389/fbioe.2023.1281503

**Published:** 2023-11-13

**Authors:** Hongtao Dai, Senlin Chai, Yao Yao, Wenlai Tang, Jianping Shi, Qing Jiang, Liya Zhu

**Affiliations:** ^1^ Jiangsu Key Laboratory of 3D Printing Equipment and Manufacturing, School of Electrical and Automation Engineering, Nanjing Normal University, Nanjing, China; ^2^ State Key Laboratory of Pharmaceutical Biotechnology, Division of Sports Medicine and Adult Reconstructive Surgery, Department of Orthopedic Surgery, Drum Tower Hospital Affiliated to Medical School of Nanjing University, Nanjing University, Nanjing, China; ^3^ Guangdong Key Laboratory of Minimally Invasive Surgical Instruments and Manufacturing Technology, School of Electromechanically Engineering, Guangdong University of Technology, Guangzhou, China

**Keywords:** intermittent pneumatic compression, deep vein thrombosis, microfludic vein chip, blood flow, numerical method

## Abstract

**Background:** Deep Vein Thrombosis (DVT) is a common disease, frequently afflicting the lower limb veins of bedridden patients. Intermittent Pneumatic Compression (IPC) is often employed as an effective solution for this problem. In our study, a random selection of 264 patients underwent IPC treatment for either one or 8 hours daily. The rate of severe venous thrombosis was substantially reduced in the IPC-treated group compared to the control group. However, real-time monitoring of blood flow during IPC operation periods remains a challenge, leading to rare awareness of IPC working mechanism on thrombosis prevention.

**Methods:** Here, microfluidic chip methodology is used to create an *in vitro* vein-mimicking platform integrating venous valves in a deformable channel. Whole blood of patients after knee surgery was perfused into the venous channel at a controlled flow rate obtained from patients with IPC treatment clinically.

**Results:** According to the numerical simulations results, both of an increase in compressive pressure and a decrease in time interval of IPC device can accelarete blood flow rate and the shear stress within the vein. The vein chip experiments also reveal that the fibrin accumulation can be greatly lowered in IPC treated group, indicating less thrombosis formation in future. A time interval of 24 seconds and a maximum contraction pressure of 40 mmHg were proved to be the most effective parameters for the IPC device adopted in our clinical trail.

**Conclusion:** This vein chip presents a novel method for observing the functional mechanisms of IPC device for DVT prevention. It provides crucial data for further standardization and optimization of IPC devices in clinical usage.

## Introduction

DVT is characterized by the abnormal formation of blood clots within deep veins, frequently occurring during the perioperative period of major orthopedic surgery (Y. Lu, Yang and Wang, 2019). DVT can lead to pain, edema, and ulcers, and in severe cases, disability and death (Wiznia et al., 2019; Schrempf and Anthuber, 2021). It is established that DVT arises from a combination of flow stasis at the cusps, hypoxia-induced activation of the endothelium, and subsequent accumulation of procoagulant factors (Lehmann et al., 2018). In clinical practice, DVT prevention can be achieved through mechanical and pharmacological measures (Dennis, Sandercock, Murray and Forbes, 2013; Long et al., 2014). Mechanical methods use Intermittent Pneumatic Compression (IPC), Graduated Compression Stockings (GCS), and venous foot pumps to enhance blood circulation (Elbuluk et al., 2018; Wall et al., 2019). Pharmacological prophylaxis incorporates aspirin, rivaroxaban, and Low Molecular Weight Heparin (LMWH) (Tamowicz, Mikstacki, Urbanek and Zawilska, 2019a; Tamowicz et al., 2019b; L; Wang et al., 2022). Although nonsteroidal anti-inflammatory drugs (NSAIDs) can reduce vascular disease, they can also heighten the risk of major bleeding (de Vries et al., 2019; D. S; Lu et al., 2015). LMWH demonstrates modest effectiveness in DVT risk reduction compared to untreated groups, which can be attributed to decreased blood flow to the limb due to immobilization of the muscle pump, a significant factor in DVT pathogenesis (Domeij Arverud et al., 2015). Mechanical measures serve to increase the volume flow rate and velocity of blood reflux in the vein without posing a risk of bleeding or burden on the liver and kidneys, especially for patients at high risk of bleeding complications (Park, Lee, Lee and Cho, 2016; Simao, Ferreira, Mora-Rodriguez and Ramos, 2016).

Orthopedic procedures such as joint arthroplasty can facilitate the entrance of fat droplets, cell clumps, and other emboli from the bone marrow cavity into the cardiovascular system, enhancing the risk of endothelial cell damage and inflammation. Postoperative bed rest and limb immobilization further heighten the risk of postoperative DVT. Since the 1960s, IPC devices have been widely utilized in human limb and joint treatments (Laupland, Tabah, Kelway and Lipman, 2019; L; Wang, Duan, Liao, Luo and Hou, 2020). For instance, Chibbaro et al. (Chibbaro et al., 2018) divided patients into two groups, A (No IPC), and B (with an IPC device). The study found that DVT, pulmonary embolism (PE), and mortality rates were lower in group B than group A. Dennis et al. (Dennis, 2014) also affirmed that IPC devices could significantly prevent thrombus formation in patients with varying conditions. Galyaev et al. (Galyaev, 2018) found a 62% reduction in DVT risk with IPC device usage, while Nandwana et al. (Nandwana and Ho, 2019) demonstrated that the sequential compression mode of IPC could significantly increase peripheral limb muscle oxygenation. Lee et al. (Lee et al., 2018) proposed a novel IPC method aimed at maintaining blood flow stability to enhance safety. The compressive force driven by IPC devices deforms the veins, yielding a desirable physiological effect in thrombus prevention (DeRoo et al., 2021; Y; Liu et al., 2022; Ren et al., 2022; X; Wang, Song, Ni, Lu and Mao, 2019). However, the majority of current studies are limited to clinical trials, and there are no established standards for the pressure and working time intervals of IPC pumps, resulting in inconsistent IPC usage in clinical practice.

Moreover, the process and mechanism of the IPC pump in mitigating lower limb DVT remain largely elusive. The advent of high-precision technologies such as Super Micro-vascular Imaging (SMI) (Hagiwara et al., 2018) and Color Doppler (Hara et al., 2019) has introduced novel research techniques. Nonetheless, it remains challenging to precisely observe venous flow patterns, blood cell trajectories, or real-time thrombus growth. Furthermore, varying flow patterns can emerge in the vein based on different compressive pressures and working time intervals. Microfluidic chip technology presents a promising approach to mimic the motion of vein blood *in vitro* (Takehara, Sakaguchi, Sekine, Okano and Shimizu, 2019; Rajeeva Pandian, Walther, Suresh, Cooke and Jain, 2020). This flexible channel, embedded with symmetrical valves, can identify the blood flow pattern generated by muscle pump compression through high-speed optical visualization equipment.

Inspired by the advantages of orgin chip, we present a vein chip which is a microfluidic model of a femoral vein including embaded vaves, where whole blood from patients was perfused across the channel. Further, a finite element analysis model of the venous vein was established, and Computational Fluid Dynamics (CFD) was employed to investigate blood behavior within the vein. Then the growth of thrombosis was analized by visualizing the fibrin and platelets accumulation in the vein chip. Finally, we determine how the maximum compressive pressure and working intervals of IPC equipment affect DVT formation. The results from our research can be used to provide usage guidance of IPC devices in clinical treatment.

## Materials and methods

### Clinical patient selection

**FIGURE 1:**
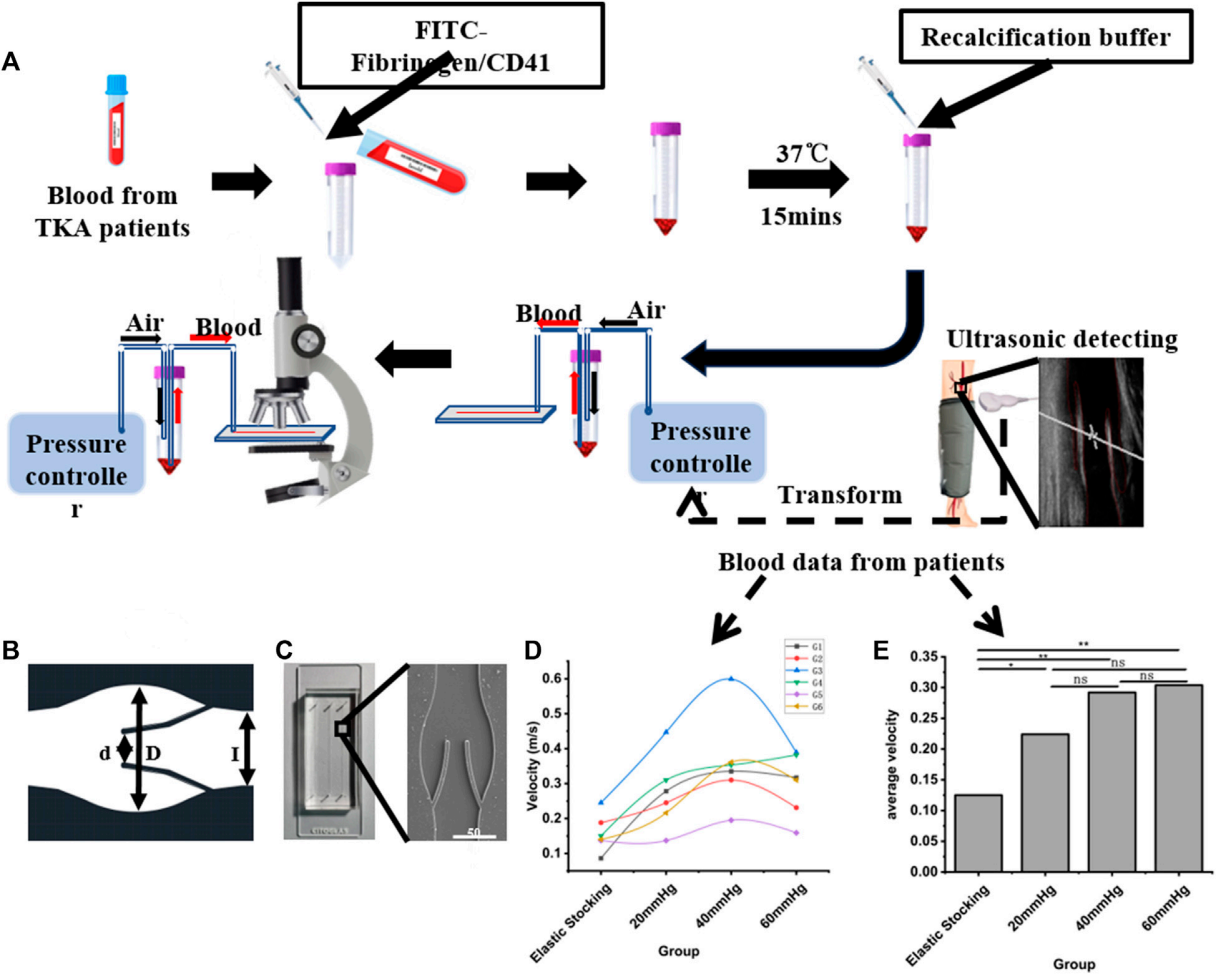
Schematic diagram of the experimental procedure. **(A)** Schematic flow diagram of microfluidic chip design, clinical data acquisition and valve area deposition experiments. **(B)** CAD figure of the vein chip. **(C)** A photograph and electron microscope view of the vein chip. **(D)** Maximum blood flow rates among patients with or without IPC. **(E)** Average blood flow rates among patients with or without IPC. *p-value < 0.05 was considered statistically significant.

This study was sanctioned by the institutional ethics committee, and consent was obtained from all patients. Exclusion criteria included: 1) patients with pre-existing conditions, such as fractures, coagulation disorders, or known lower limb VTE prior to admission, and 2) patients with incomplete information for any other reason. From September 2013 to August 2015, a total of 543 patients undergoing arthroplasty were included in the clinical data collection. After applying the exclusion criteria, 278 patients were removed from the study. Ultimately, 264 patients were included in the study. From September 2013 to July 2014, patients who used the pneumatic pump for 1 hour daily until discharge were categorized into the control group. From February 2015 to August 2015, patients who used the pneumatic pump for 24 h on the first postoperative day, followed by 8 hours daily until discharge, were categorized into the IPC group. Patients’ general condition and thrombosis diagnosis were collected. This part of the work was a retrospective study following the Declaration of Helsinki and was approved by the Ethics Committee of Nanjing Drum Tower Hospital (Ethics Number: 2012029). We performed Color Doppler ultrasound on the femoral vein of both the surgical and healthy sides (Supplementary Figure S4), maintaining a supine position during the operation to negate the impact of posture on femoral flow velocity and ensure the patient’s steady breathing and silence. Subsequently, we measured the vein velocity, monitoring patients of diverse ages and genders after knee or hip replacement surgery. We observed no significant difference in femoral vein blood flow velocity between the surgical and healthy sides. As illustrated in Figures 1D, E, both the maximum and average blood flow velocities were higher with IPC at maximum pressure values of 40 mmHg or 60 mmHg. The data also indicated that patients with initially slow basal flow rates might exhibit a higher peak flow rate with IPC.

### Finite element analysis

Doppler ultrasound image scans were performed with patients in a relaxed supine position, and the blood flow velocity was measured at a point between the venous valves of the femoral vein. The mass and momentum equations were solved using COMSOL Multiphysics (v5.5) software based on finite element analysis. The geometric model of the vein consisted of two pairs of valves and a long Micro-floppies channel originating from the patient’s veins. Valve leaflets were treated as linear elastic materials with a Young’s modulus of 100 kPa (Gupta, Zhao and Evans, 2019; Schofield et al., 2020). Blood was considered as a non-Newtonian fluid with a constant density of 1,050 kg/m^3^ and a Reynolds number of 1.05, approximating human blood characteristics. Blood flow was treated as an incompressible fluid flow. The veins were periodically compressed by the IPC device, including the time for one working cycle and the time interval between two working cycles. During the working cycle, the inlet boundary condition was adapted with piecewise functions based on clinically measured data. In the interval period, the blood would flow smoothly in the vein channel. The valve leaflets were constrained to the vein wall.

Poiseuille’s law was employed to describe the laminar pressure confined to the vein channel. The blood flow rate was generated by pressure differences at two points in the vein. The relationship between blood flow velocity ( v ) and the radius (*r*) can be expressed as (Westerhof, Stergiopulos, Noble and Westerhof, 2019):

$$v=\frac{p_{1}-p_{2}}{4\eta \iota }\left(R^{2}-r^{2}\right)$$ (1)

where, *l, p*_1_ *-p*_2_ *, η,* and *R* represent the length of the vein channel, the pressure gradient between the two ends of the vein channel, and the inner radius of the vein channel, respectively.

### Design and fabrication of vein chip

To acquire the anatomy architecture of human vein, we analyzed Doppler-ultrasound images of the femoral vein using a Portable Color Doppler (PCD, M-turbo, SONOSITE Co.) (Figure 1A). Then the geometric parameters of the vein chip was designed. Considering the maximum width of the venous valve, and the distance between the valve leaf tip and the valve bulb, the channel dimensions were set as follows: The channel width (*L*) and depth were designated as 350 μm and 50 μm, respectively, with a gap width (*d*) between the leaflets of 160 μm (Figure 1B). The venous valve bulb width was set at 600 μm. The vein chip model was designed using CAD software (Autodesk). The body of the vein chip was constructed by etching channels into PDMS using soft lithography technology. These PDMS blocks featured three parallel channels per chip, each with an inlet and outlet hole of 1.0 mm in width. The opening of each channel was approximately 50% of the channel’s width. Plasma bonding was utilized to bond PDMS and slides together. Following these specifications, we fabricated vein chips each including three channels with 40 mm length and 5 mm intervals (Figure 1C and Supplementary Figure S3).

### Blood sample collection and testing

5 mL blood sample was obtained from the median cubital vein of patients the day following total joint arthroplasty. This was stored in a vacuum tube containing 3.2% sodium citrate to prevent coagulation. Informed consent was obtained from all donors for the collection and usage of blood samples. FITC-labeled human fibrinogen (at a final concentration of 12.5 μg/mL) was incorporated into the collected blood to illuminate fibrin deposition. Platelets were stained by FITC-labeled CD41 Monoclonal Antibody (final concentration: 5 μg/mL). The labeled blood was incubated at 37°C for 15 min before the assay, enabling the FITC to yield a sharper fluorescence under blue light excitation. The prepared blood sample was quickly combined with a recalcification buffer (75 mM CaCl_2_ in PBS) to restore coagulation. Prior to infusing blood into the vein chip, the channel was primed with a PBS solution containing 2% bovine serum albumin (BSA). Venous thrombosis progression was assessed via fluorescence microscopy (IX83, Olympus Co.).

Venous blood was centrifuged at 3,000 rpm for 15 min. The resultant red blood cells (RBCs) were then introduced into a PBS solution to achieve a hematocrit value of 0.5%. Under an inverted microscope, RBCs served directly as tracer particles, illustrating the flow field in a bright field. The motion of the RBCs, under the gradient pressure of the controller (Elvesys Co.), was documented using a high-speed camera (M110, Phantom Co.). The captured footage was examined to determine the fluid velocity field (Supplementary Figure S1). The high-speed camera operated at a capture rate of 2000 frames per second. Blood flow velocities within the veins at different IPC pressures were derived from preliminary clinical data. During observations of venous thrombosis, console parameters were adjusted to ensure the blood flow velocity in the Micro-floppies channel corresponded with the venous blood flow velocity.

### Blood flow velocity measurement

Utilizing an inverted microscope (CKX41, Olympus Co.), the channel width served as the standard length for gauging the velocity of Red Blood Cells (RBCs) within a bright field. The flow of RBCs was captured with a high-speed camera (M220, Qian Yanlang Co.) operating at 2,000 frames per second (fps). However, synchronous measurement of blood velocity during flow presented a challenge. Multiple measurements were taken and averaged to overcome this issue. Experimental trials were conducted under four conditions: patients wearing only elastic stockings, and patients with IPC under pressures of 20, 40, and 60 mmHg. Cycle periods were set at 7.2, 8.2, and 9.8 s, corresponding respectively to the pressures of 20, 40, and 60 mmHg. In the end, distinct RBCs were selected as velocity measurement points, and the average RBC speed was calculated (Supplementary Figure S1). More original data was uploaded in the Supplementary Materials. If any readers are interested in further details, they can contact 61193@njnu.edu.cn.

## Results

### Clinical utility analysis

In this study, a cohort of 264 patients was assessed. Among these, 135 patients utilizing the pneumatic pump for 1 h daily until discharge were assigned to the control group. The remaining 129 patients, included in the pump group, used the pneumatic pump continuously for 24 h on the first postoperative day, and then for 8 h daily until discharge. The mean ages of the control and IPC groups were 63.79 ± 11.49 years and 64.74 ± 10.55 years, respectively. The average D-dimer levels for the control and IPC groups before surgery were respectively 0.95 ± 1.16 and 1.05 ± 1.76 (Supplementary Figure S2). Statistical analysis revealed no significant difference between the two groups (*p >* 0.05). Detailed data are presented in Table 1.

**TABLE 1:** Basic clinical characteristics of patients participating in this study.

Variables	Control (Mean ± SD)	IPC (Mean ± SD)	*p*-value
Age (years)	63.78 ± 11.49	64.74 ± 10.55	*p* = 0.485
BMI (kg/m^2^)	25.14 ± 4.01	25.81 ± 3.87	*p* = 0.173
Gender (female)	90 (66.7%)	96 (74.4%)	*p* = 0.169
Fbg (g/L)	3.00 ± 0.60	2.97 ± 0.73	*p* = 0.378
D-dimer (mg/L)	0.95 ± 1.16	1.05 ± 1.76	*p* = 0.468

In the control group, DVT was identified in 27 patients (20.0%), as confirmed by venography and ultrasound findings. This group showed the occurrence of Soleal Vein (SV) thrombosis in 22 patients (16.3%), Peroneal Vein (PEV) thrombosis in four (3.0%), Posterior Tibial Vein (PTV) thrombosis in two (1.5%), and Femoral Vein (FV) thrombosis in one (0.7%). There was one case of combined PTV, PEV, and SV thrombosis. In contrast, within the IPC group, 19 patients (14.7%) were diagnosed with DVT, including 18 cases (14.6%) of SV thrombosis and one case (0.8%) of PTV thrombosis. The incidence of DVT was not significantly different between the two groups (*p* = 0.326). However, the frequency of major venous thrombosis (comprising PEV, PTV, and FV) was notably lower in the IPC group than in the control group (*p* < 0.05). Table 2 illustrates the specific distribution of thrombosis cases. On the day of venography, plasma D-dimer levels registered at 3.34 ± 1.94 mg/L for the control group, and 2.53 ± 1.68 mg/L for the IPC group. Given the established correlation between high D-dimer levels and increased thrombosis risk, these findings suggest that the IPC method can effectively reduce the risk of thrombosis.

**TABLE 2:** Distribution of different kinds of DVT among the patients.

Variables	Control N (%)	IPC N (%)	*p*-value
DVT	27 (20.0%)	19 (14.7%)	0.326
SV	22 (16.3%)	18 (13.9%)	0.596
PEV	4 (3.0%)	0 (0%)	—
PTV	2 (1.5%)	1 (0.8%)	—
FV	1 (0.7%)	0 (0%)	—
PEV + PTV + FV	7 (5.2%)	1 (0.8%)	0.048

### CFD analysis of vein chip

To analyze the effect of various IPC treatments, we classified clinical patients into four groups: NIPC (no treatment with IPC), and those with 20 mmHg, 40 mmHg, and 60 mmHg IPC (corresponding to the maximum compressive pressure of IPC). Both the NIPC and IPC groups wore elastic stockings on their lower limbs during the experiment. We conducted CFD techniques on the model of the femoral vein, with the blood flow rate modeled using mathematical functions that emulate the clinical scenario. The maximum velocities for the IPC groups (20, 40, and 60 mmHg) were 1.6, 1.7, and 1.8 times those of the NIPC group, respectively. Subsequently, we studied the shear stress and laminar pressure within the vein.

**FIGURE 2:**
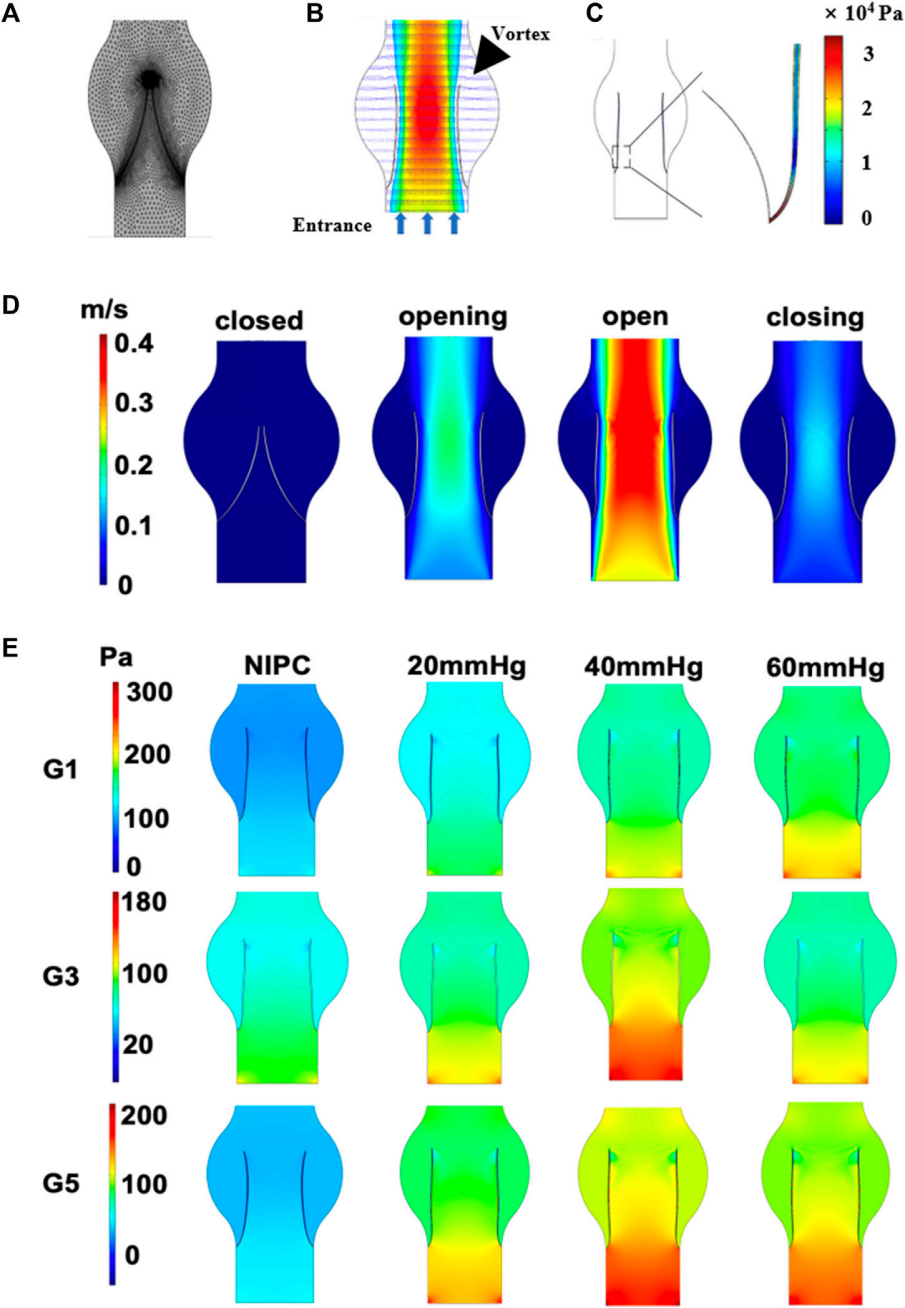
Contour maps illustrating pressure and velocity derived from CFD results. **(A)** Mesh elements of the vein channel. **(B)** Velocity contours around the valve. **(C)** The shear stress on one side of the venous valves. **(D)** Variation in velocity as leaflets open over time. **(E)** Laminar flow pressure within the vein channel.

The behavior of blood flow around the valve and its pattern are presented in Figure 2A. Through the main channel, a high-speed jet is generated. Concurrently, two contra-rotating vortices can be observed at the cusp of the venous valve. Figure 2B depicts the distribution of shear stress around the venous valve leaflet when the valve is fully open. The peak shear stress occurs at the point where the valve connects to the wall and then decreases towards the tip of the valve leaflet. The reflux can reach the base of the pocket and expand the flow range in this region. The variation in flow pattern as the leaflets open over time is shown in Figure 2C. Initially, the valve is closed, and there is no blood flow in the channel. However, as the inlet velocity increases, the blood velocity also rises, passing the valve and flowing towards the outlet. The degree of valve opening increases in response to the escalating velocity. The velocity reaches its maximum magnitude where the cross-sectional area between the vein channel walls is the smallest.

Laminar flow is characterized by concentric layers of blood moving in parallel along the vein. Figure 2D and Supplementary Figure S5 separately presents the steady flow conditions for patients G1, G3, G5 and G2, G4, G6 when the valve is also fully open. The highest laminar flow pressure is detected in the center of the vein channel, while the lowest velocity is found along the channel wall. The distribution of the laminar flow pressure aligns with that of the flow velocity. A parabolic flow pattern is scarcely seen in the control group. In contrast, a steady laminar flow is observed across all the IPC groups. The blood flow below 40 mmHg displays the highest value of laminar flow pressure. No significant difference is noted for patient G3 under IPC treatment at 20 and 60 mmHg.

### Growth of fibrinogen and platelets deposition

**FIGURE 3:**
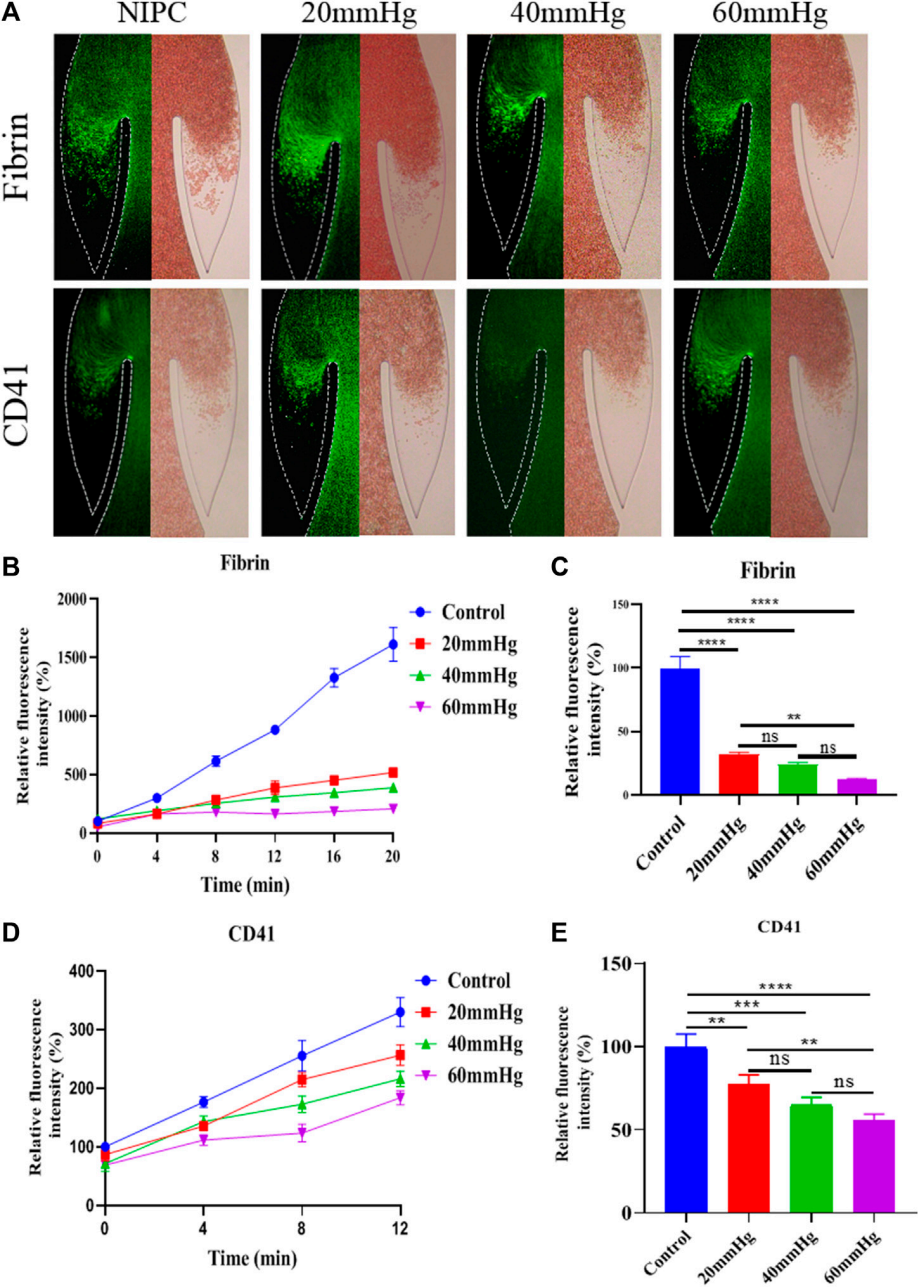
Progression of fibrinogen and platelets deposition in altered blood flow under various IPC pressures. **(A)** Fluorescence micrographs of vein chips after perfusion of blood labeled with fibrin (top), platelets (bottom) for control and different IPC groups. **(B)** Relative intensity of fibrin over 20 min, and **(C)** at the 12th minute. **(D)** Relative intensity of platelets over 20 min, and **(E)** at the 12th minute. *p-value < 0.05 was considered statistically significant.

Modes Platelets and fibrins are often used as markers to visualize the formation of thrombus. The accumulation of thrombus in the valve pocket was examined by using fluorescence microscopy to observe the deposition of fibrinogen and platelets. We analyzed the growth of thrombus over 20 min in the valve pocket, both with and without the application of various maximum IPC pressure levels. The IPC device operated consistently with a time interval of 48 s. As demonstrated in Figure 3A, Red Blood Cells (RBCs), platelets, and fibrin began to accumulate after 20 min, especially in the valve pocket. The number of fibrin molecules in the vortex zone was significantly lower in all IPC groups than in the control group (*p* < 0.01). Fibrin accumulation decreased with increased IPC pressure (Figures 3B, C). Higher IPC pressures also resulted in a decrease in platelet adhesion within the valve pocket (Figures 3D, E). It can be concluded that the altered blood flow induced by IPC had an impact on thrombus formation in venous valves. Both the blood flow rate and the shear stress increased as the IPC pressure rose. Even though higher shear stress might activate more platelets (Rajeeva Pandian et al., 2020), fibrin monomers and oligomers became unstable, thereby still reducing fibrinogen and platelets deposition. Simultaneously, stagnation of blood flow might trigger the release of inflammatory factors, potentially further increasing fibrinogen and platelets deposition.

**FIGURE 4:**
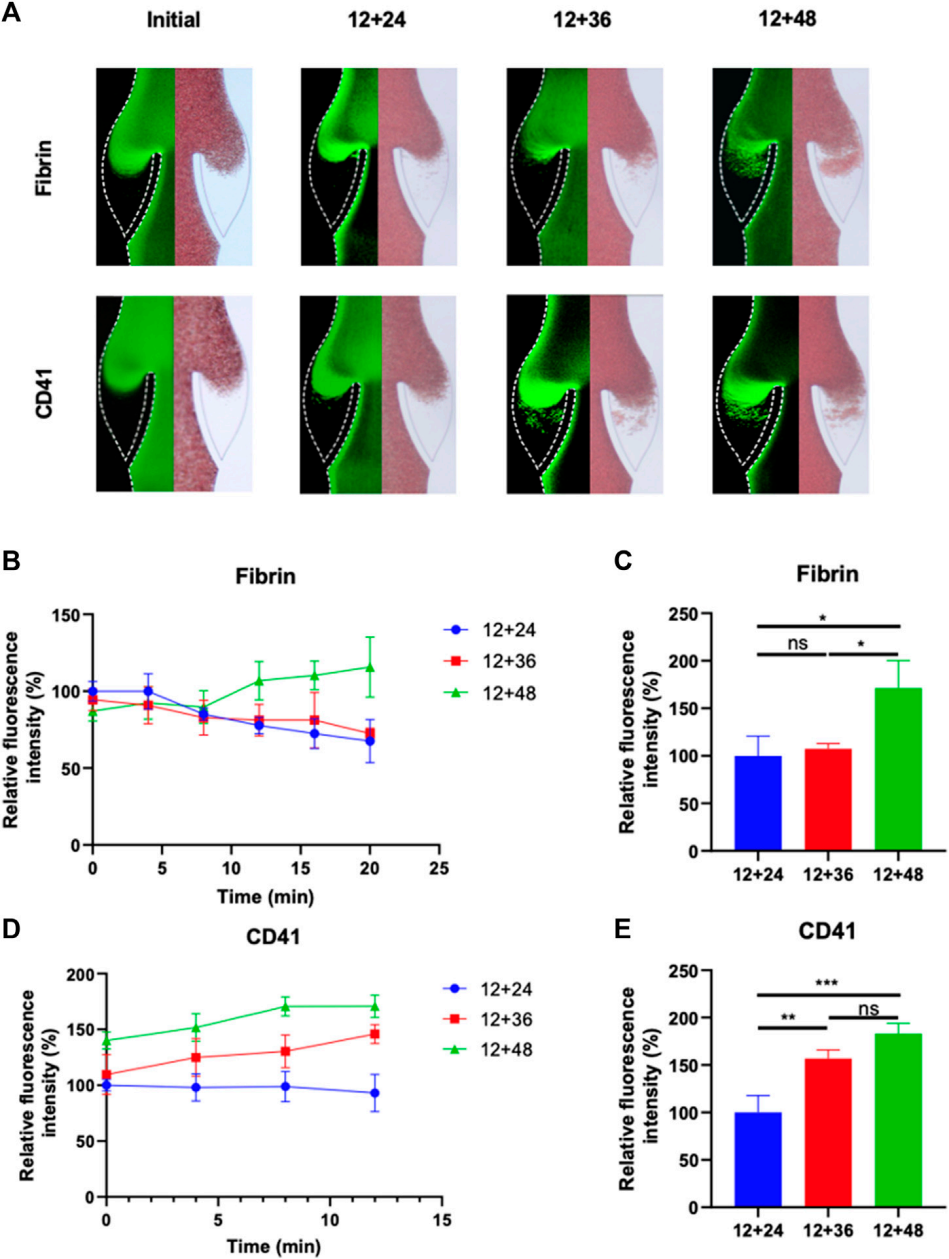
Progression of fibrinogen and platelets deposition in altered blood flow under various IPC time intervals. **(A)** Fluorescence micrographs of vein chips after perfusion of blood labeled with fibrin (top), platelets (bottom) for control and different IPC groups. **(B)** Relative intensity of fibrin over 20 min, and **(C)** at the 12th minute. **(D)** Relative intensity of platelets over 20 min, and **(E)** at the 12th minute. *p-value < 0.05 was considered statistically significant.

The growth of thrombus over a 20-min period in the valve pocket, with IPC treatment at varying time intervals, was also analyzed (Figure 4A). The IPC device functioned with a constant working time of 12 s and different interval times of 24, 48 and 60 s. Initially, a small area of deposition was observed in the valve pocket. Deposition of fibrinogen and platelets increased with the expansion of the interval time. The accumulation of fibrin and platelets in the valve at the 12 nd min was approximately double for an interval time of 60 s compared to an interval time of 24 s (Figures 4C, E). No significant difference was observed between the 12 + 24 and 12 + 48 groups in terms of fibrinogen deposition reduction (*p* > 0.05). Conversely, a substantial difference was found between the 12 + 24 and 12 + 48 groups regarding platelet deposition reduction in the valve area (*p* < 0.01). These findings suggest that the altered blood flow caused by longer IPC interval times is more influential on platelet accumulation than on fibrin. An IPC device with a working time of 12 s and an interval time of 24 s was considered the most optimal setting for preventing fibrinogen and platelets deposition.

## Discussion

DVT frequently occurs in the lower limb veins of patients confined to bed following hip or knee replacement surgery (Ruban, Yeo, Seow, Tan and Ng, 2000). Trunk vein thrombosis can result in severe conditions such as pulmonary embolism. We have conducted numerous analyses of thrombus formation in patients receiving IPC treatment post-knee surgery. This preventive measure, which mimics natural muscle pumps, has been shown to reduce the incidence of DVT. However, different studies have used different IPC pump pressures, times, and sites, and there is a lack of cross-validation (Supplementary Table S1). Moreover, few *in vitro* studies have been reported on how IPC affects venous blood flow in the lower extremities. In this study, we developed a Micro-floppies chip system, simulating a human lower limb vein, to observe venous thrombosis progression in real time and assess differences in thrombosis under various IPC parameters. The microchip shares structural similarities with the valve region of deep veins in the lower limb and can simulate flow in the valve region, where thrombosis most frequently occurs. Additionally, the blood flow rates in the Micro-floppies chip were derived from clinical data from patients’ lower limbs. By integrating data simulation with actual measurements, we anticipated that the microchip system would facilitate analysis of the impact of various IPC working parameters on venous thrombosis formation. The possibility of thrombosis occurrence under different IPC working parameters were then concluded from both numerical and experimental results.

The study examined the compressive pressure amplitude and frequency of the IPC device. The maximum pressure ranged from 20 to 60 mmHg. The working time was consistently set at 12 s for all IPC groups, but the time intervals varied: 24, 48 and 60 s, in line with the commercial device. Immobility often results in low-velocity blood flow and constrained blood exchange within the valve pocket. Without IPC compression, it was observed that blood stasis in the valve pocket was likely to form. When the IPC operated periodically, it generated a vortex in the valve pocket. The velocity peaked as it passed the valve and decreased along the walls, corresponding to the decrease in the flow cross-section area, and *vice versa*. Despite visible vortices behind the leaflets, their velocities were lower, especially near the junction of the valve and the vein wall. The vortices became more pronounced towards the valve tips.

Mobile clots can be propelled by blood flow. With higher compressive pressure or shorter time intervals between working cycles, increased flow velocity could reduce fibrin and platelet deposition in the valve. Without sufficient shear stress and flow velocity, blood stasis could lead to thrombus formation. As compressive pressure decreased or time intervals lengthened, the vortex was incapable of disrupting the network composed of cross-linked fibrin, aggregated platelets, and red blood cells (Singh, Houng and Reed, 2019). The results demonstrated that high blood flow pressure curtailed fibrin linkage, whereas shorter time intervals controlled platelet recruitment. By incorporating venous valves and blood flow into this vein chip, we discerned optimal working parameters for IPC devices, which could inform the design of future therapeutic methods.

Nonetheless, the vein chip platform presents several limitations to consider in future work. The blood samples used were stored in sodium citrate for anticoagulation and recalcified for use, which could alter platelet function due to excessive calcium (Colace, Tormoen, McCarty and Diamond, 2013). We also observed that thrombus formation was quicker in the microengineered vein chip than in actual clinical veins, possibly due to the absence of two critical factors in thrombus formation: endothelial dysfunction and hypercoagulation. The vein’s endothelial cells significantly influence blood cell adhesion, vascular inflammation, and thrombosis formation (Welsh et al., 2019). Hypoxic conditions in the chip channel might also trigger the release of inflammatory mediators that could enhance thrombus formation (Gutmann, Siow, Gwozdz, Saha and Smith, 2020). Furthermore, the valve leaflets on this chip were fixed due to manufacturing technology limitations (Ariane et al., 2017; Hajati, Sadegh Moghanlou, Vajdi, Razavi and Matin, 2020; X; Liu and Liu, 2019; Martinod and Wagner, 2014). However, natural venous valves are flexible, permitting increased blood flow through the vein when open. The mechanical modulus, gap distance, and leaflet shape also affect cell or protein attachment (Schofield et al., 2020).

## Conclusion

In summary, this microfluidic vein chip initially observed the influence of working mechanism of IPC equipments on DVT prevention *in vitro*. Utilizing this device, we could depict how vein valves respond to the mechanical compression and how altered blood flow impacts thrombus formation. This research provides a novel method to obtain best operation parameters of IPC devices in clinical trails. It is possible to establish mechanical treatment standards for postoperative or long-term bedridden patients in the future.

## Data Availability

The original contributions presented in the study are included in the article/Supplementary Material, further inquiries can be directed to the corresponding authors.
