# Supplementary material for: Effect of intermittent pneumatic compression on preventing deep vein thrombosis using microfluidic vein chip
Source: Front Bioeng Biotechnol. 2023 Nov 13;11:1281503. doi: 10.3389/fbioe.2023.1281503 (PMC10679410; doi:10.3389/fbioe.2023.1281503)
Supplement: Supplementary file 1 [file Table1.docx]

**TABLE S1** Comparative analysis between this study and other similar research where IPC mitigated DVT following arthroplasty. (N/A=not available)

|  | **This Study** | **Kim, 2019** | **Liu, 2017** | **Jo, 2016** | **Tsuda, 2015** |
| --- | --- | --- | --- | --- | --- |
| Surgery Type | THA+TKA | TKA | TKA | THA | THA |
| IPC Application Pressure | 40mmHg | N/A | 45 mmHg | 30-45mmHg | 110mmHg |
| IPC Application Time | 5 days | 6 days | 48 hours | 2 days | 1-2 days |
| IPC Application Site | Calf | Ankle to thigh | Calf and thigh | Ankle to thigh | Foot |
| Additional Measures | Anticoagulants | Compression stockings | Anticoagulants | Anti-embolic stockings | N/A |
| DVT Detection after Operation | 5 days | 6 days | 9 days | 5 days | 3, 21 days |
| Number of Participants | 129 | 425 | 60 | 741 | 166 |
| Incidence of overall DVT (%) | 14.7 | 11.3 | 8.3 | 0.4 | 6.0 |
| Proximal DVT (%) | 0 | 0.9 | 0 | N/A | 0 |
| Distal DVT (%) | 0.8 | N/A | 5 | N/A | 6 |
| Intermuscular DVT (%) | 13.9 | N/A | 13.3% | N/A | N/A |
