# Supplementary material for: Effect of intermittent pneumatic compression on preventing deep vein thrombosis using microfluidic vein chip
Source: Front Bioeng Biotechnol. 2023 Nov 13;11:1281503. doi: 10.3389/fbioe.2023.1281503 (PMC10679410; doi:10.3389/fbioe.2023.1281503)
Supplement: Supplementary file 2 [file DataSheet1.docx]

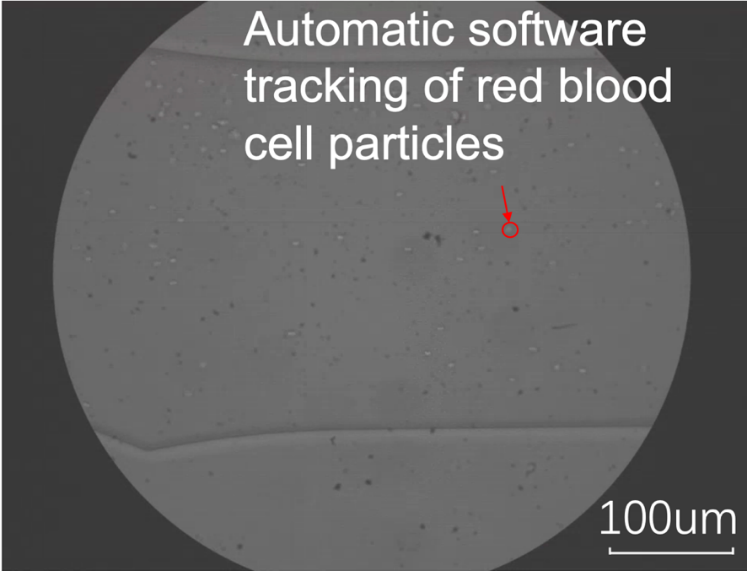


**FIGURE S1** Captured images of red blood cell for velocity measurement.


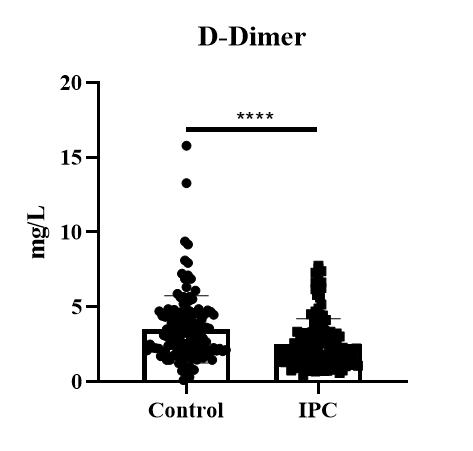


**FIGURE S2** D-dimer levels of blood collected from the control and IPC group patients.


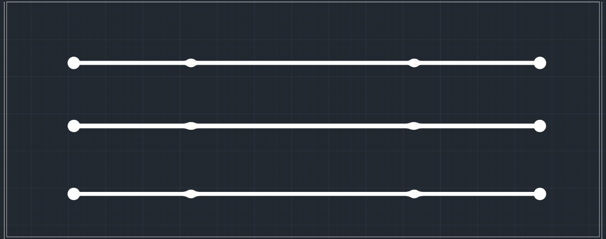


**FIGURE S3** CAD drawings of vein chips integrated three channels.


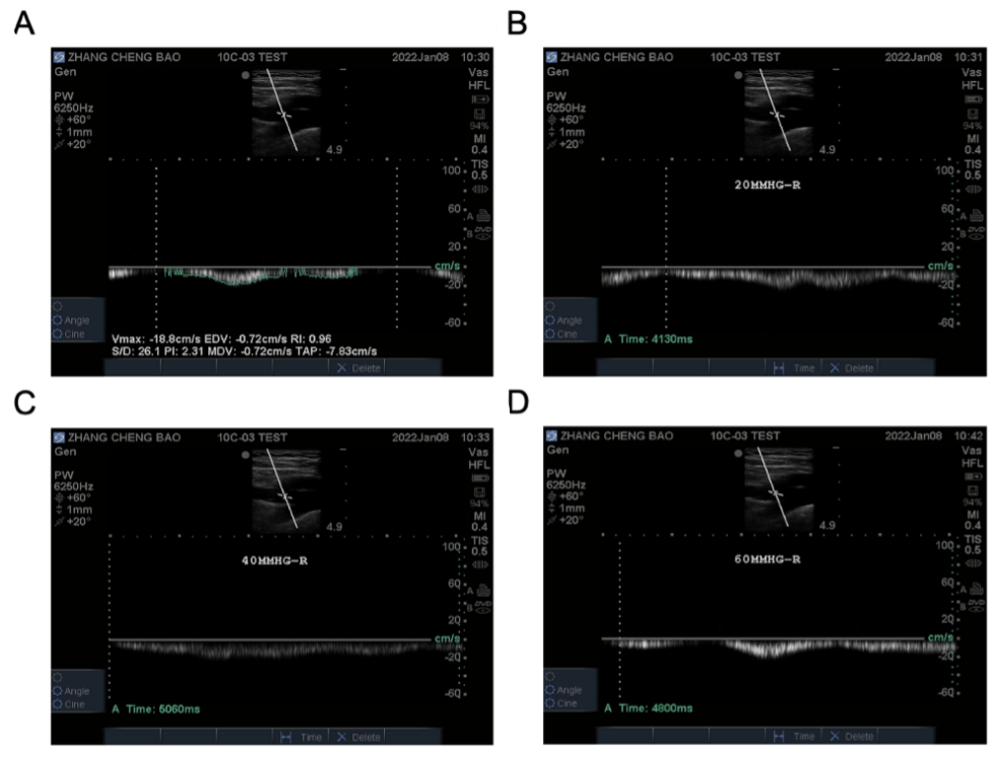


**FIGURE S4** Doppler ultrasound images of the femoral vein. **A** Patients only wearing the elastic stockings. Patients taking IPC treatment with the maximum pressure of **B** 20mmHg **C** 40mmHg and **D** 60mmHg.


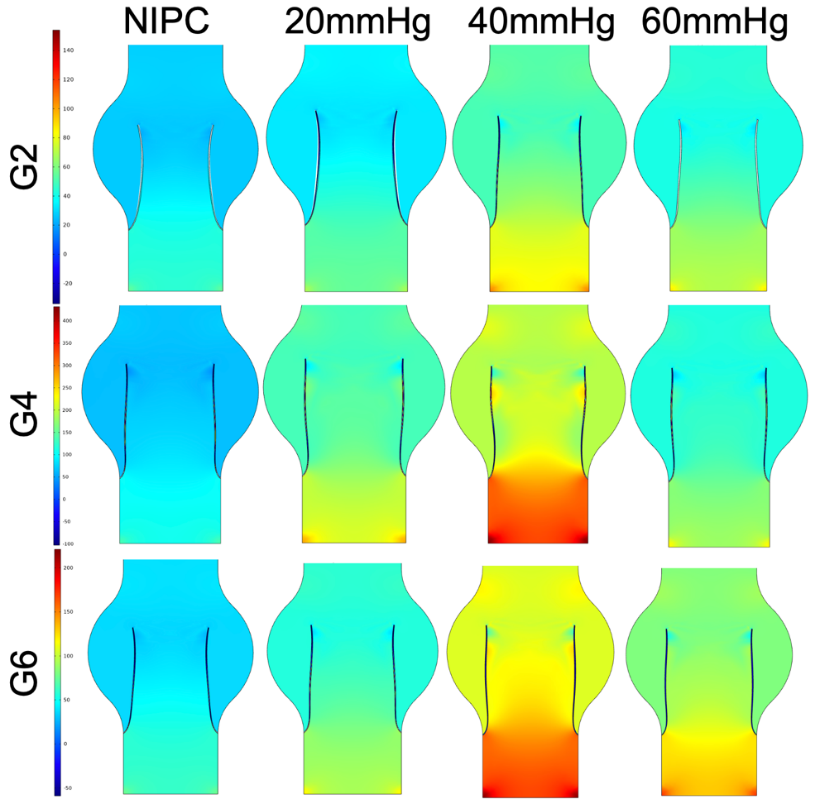


**FIGURE S5** Simulation of laminar flow pressure within the vein channel of patient G2, G4 and G6.
